# Smoking cessation policy and treatments derived from the protective motivation of smokers: a study on graphic health warning labels

**DOI:** 10.3389/fpsyg.2023.1205321

**Published:** 2023-10-31

**Authors:** Qiwei Pang, Lu Wang, Jinge Yao, Kum Fai Yuen, Miao Su, Mingjie Fang

**Affiliations:** ^1^College of International Economics and Trade, Ningbo University of Finance and Economics, Ningbo, Zhejiang, China; ^2^Department of Economics, Sejong University, Seoul, Republic of Korea; ^3^School of Business, Hangzhou City University, Hangzhou, Zhejiang, China; ^4^School of Management, Zhejiang University, Hangzhou, Zhejiang, China; ^5^College of Wealth Management, Ningbo University of Finance and Economics, Ningbo, Zhejiang, China; ^6^School of Civil and Environmental Engineering, Nanyang Technological University, Singapore, Singapore; ^7^The Graduate School of Technology Management, Kyunghee University, Yongin-si, Republic of Korea; ^8^Department of Logistics, Service and Operations Management, Korea University Business School, Seoul, Republic of Korea

**Keywords:** graphic health warning labels, health anxiety, planned behavior, protection motivation, quit smoking

## Abstract

**Introduction:**

Smoking is a leading public health risk. Many countries are reducing the demand for tobacco through graphic health warning labels (GHWLs). This study aims to explore smokers’ perceptions of GHWLs and analyze the effect of GHWLs on their behavioral intentions to quit smoking.

**Methods:**

A theoretical model is designed by synthesizing protection motivation theory, an extension of the extended parallel process model, and the theory of planned behavior. We collected a cross-sectional sample of 547 anonymous smokers through a stratified random sampling strategy. GHWLs published in 2011 by the US Food and Drug Administration were used in the survey to assess smokers’ responses to them, and then the hypotheses are validated through structural equation models.

**Results:**

The results suggest that perceived severity, perceived vulnerability, response efficacy, and health anxiety have a significant impact on smokers’ protection motivation. Furthermore, smokers’ protection motivation directly impacts the behavioral intention to quit smoking and indirectly influences intention to quit through attitudes.

**Discussion:**

These findings have practical implications for the implementation and improvement of GHWLs policies. Meanwhile, this study enriches the literature on public health protection measures (i.e., GHWLs) and smokers’ behavioral intention to quit smoking.

## 1. Introduction

Research on tobacco hazards has found that smoking causes numerous diseases, such as chronic lung disease and lung cancer and oral squamous cell carcinoma (U.S. Department of Health and Human Services, 2014; Gottfredson, 2021). Smoking and other tobacco use are the leading cause of preventable death in both developed and developing countries worldwide, responsible for approximately six million deaths per year (Wu et al., 2015; Wang Z. Y. et al., 2019). The World Health Organization (WHO) introduced the Framework Convention on Tobacco Control (FCTC) in 2003 to combat smoking (Rees, 2019; Monshi and Ibrahim, 2021). The FCTC’s measures target the demand for tobacco by raising public awareness about tobacco’s harmful effects, including graphic health warning labels (GHWLs) that can enable smokers to see the risks of smoking (Wu et al., 2015).

**FIGURE 1:**
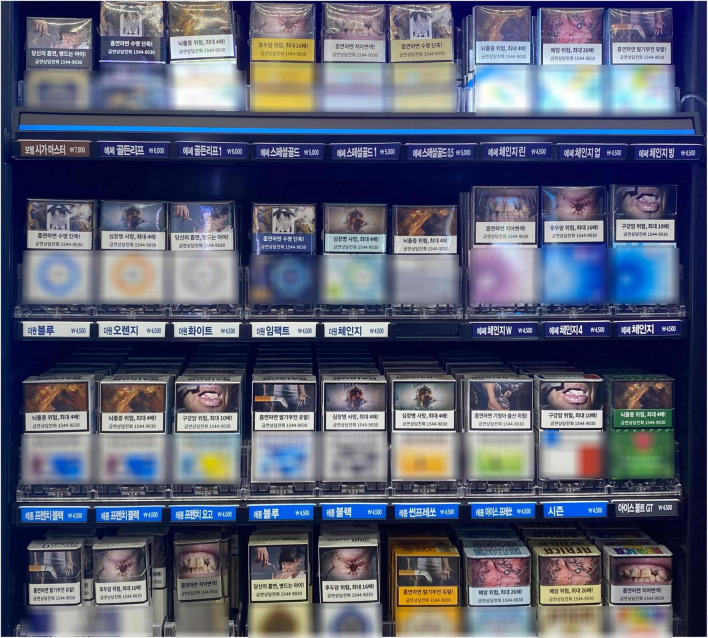
A sample of GHWLs on cigarette packs in Republic of Korea.

Because GHWLs can reduce the demand for tobacco, various countries are protecting the environment and improving health awareness using this implementation strategy. In 2009, the U.S. introduced the Family Smoking Prevention and Tobacco Control Act, requiring the U.S. Food and Drug Administration to create GHWLs for cigarettes to be placed on all cigarette packages and advertisements in the U.S. (Evans et al., 2017). In 2016, Korea also implemented this policy for the first time and required that GHWLs and text warnings cover 50% of packs (see Figure 1; Hwang et al., 2020). GHWLs currently cover almost 4.7 billion people in 101 countries, more than half of the world’s population (60%), and more than half of all countries (World Health Organization [WHO], 2021).

As the GHWLs policy is implemented more widely and governments of various countries attach importance to the public health, research on GHWLs has been surging. These studies include media coverage and smokers’ attitudes toward GHWLs. For example, by collating media coverage and other methods, Miller et al. (2009) found that 85% of media coverage had a positive attitude toward GHWLs in Australia and that GHWLs caused more than half of smokers to feel an increased risk of dying from a smoking-related illness. Grigaliunaite and Pileliene (2017) found that GHWLs can cause smokers to reinforce negative perceptions of smoking.

Furthermore, many existing studies have also found that GHWLs have a significant impact on reducing smokers’ satisfaction (Romer et al., 2018), reducing the needs of cigarettes (Thrasher et al., 2011), intention to quit smoking (Verlhiac et al., 2011; Wu et al., 2015; Evans et al., 2017) and preventing smoking initiation (Villanti et al., 2014). Unfortunately, few studies have systematically explored the antecedents of these results, with insufficient research on the psychological activities for which smokers have intrinsic protective motivation (Rogers, 1983) and health anxiety (So, 2013) after seeing GHWLs, triggering their intention to quit smoking.

However, by studying smokers’ protective motivation and health anxiety, establishing targeted behavior change research models can better reveal the psychological effects of GHWLs on smokers (Floyd et al., 2000; Sabzmakan et al., 2018). Such studies can better formulate the necessary, related strategies (Boss et al., 2015) to reduce the impact of tobacco on public health and the environment.

Therefore, the purpose of this study is to develop a targeted research model to fill the knowledge gap in this field by determining the motivations and reasons for smokers choosing to quit smoking after seeing GHWLs. This study is based on protection motivation theory (PMT) (Rogers, 1983) combined with an extension of the extended parallel process model (E-EPPM) (So, 2013) and the theory of planned behavior (TPB) (Armitage and Conner, 2001). PMT was chosen because it provides a unique framework for predicting health behaviors (Roozbahani et al., 2020) that can analyze smokers’ potential psychological factors (e.g., perceived vulnerability; perceived severity; response efficacy; protection motivation) (Rogers, 1983) associated with quitting smoking after seeing the risk from GHWLs.

However, smokers are generally prone to smoking for a long time (Robinson et al., 2020). When they know that this behavior is unhealthy, it causes them anxiety (Haaga et al., 2020). The anxiety, which PMT does not consider, must be analyzed by combining PMT with E-EPPM (e.g., health anxiety). Furthermore, TPB posits that attitudes will affect behavioral intentions (Pang et al., 2021). This theory has been proposed to be appropriate for quitting smoking (Sabzmakan et al., 2020).

This study was conducted in China, with more than 300 million smokers (ranked first in the world) (Mak et al., 2020; Liu et al., 2021), but Chinese smokers are not protected by GHWLs. Surprisingly, instead of GHWLs, cigarette packages in China have been well designed with attracting slogans and pictures (Wu et al., 2015). We assert that collecting the attitudes of Chinese smokers is meaningful for reducing tobacco use worldwide. Simultaneously, analyzing the attitudes and intentions of Chinese smokers after they see GHWLs can reveal empirical evidence for whether to implement a GHWLs-related policy in China.

The remainder of this paper is structured as follows. Guided by these current theories, we present the research model and hypotheses in section “2. Literature review and hypothesis development.” Section “3. Materials and methods” describes the survey design and research methodology. Section “4. Results” presents the data analysis and the results of the hypotheses, while section “5. Discussion” summarizes the implications of this study, limitations, and directions of future research.

## 2. Literature review and hypothesis development

### 2.1. Theoretical background

**FIGURE 2:**
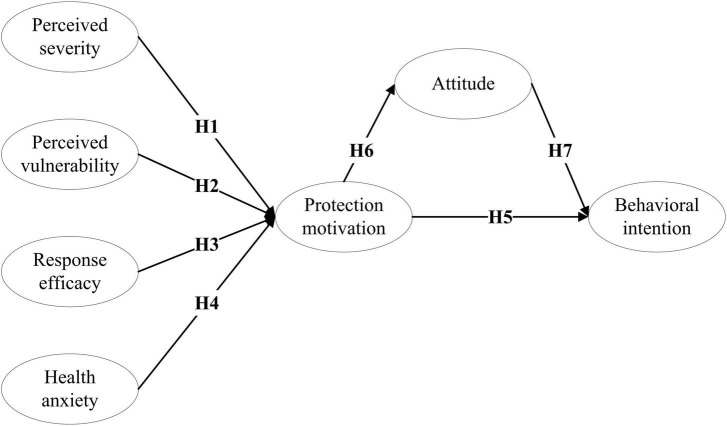
Research model.

This study adopts PMT, E-EPPM, and TPB to present the research model (Figure 2), analyzing how smokers’ intention to quit smoking is influenced by their protection motivation, health anxiety, and attitude. Table 1 describes the paradigm, basic assumptions, key constructs, and model applications of each theory.

**TABLE 1:** Theories explaining smokers’ behavioral intention to quit smoking.

Theory’s characteristics	Protection motivation theory	The extension of the extended parallel process model	The theory of planned behavior
Paradigm	Psychology	Psychology	Psychology
Basic assumption	Behavioral intention to protective health is influenced by PMT constructs.	Risk avoidance behavior is influenced by cognitive components and affective components.	Human behavior is the result of a well-thought-out plan.
Key constructs	Protection motivation, perceived severity, perceived vulnerability, response efficacy	Anxiety, fear, uncertainty, severity	Behavioral intention, attitude, subjective norm, perceived behavioral intention
Application to model	Theory explains how the structures of PMT affect the protection behavior and attitudes of smokers after seeing GHWLs.	Theory explains how smokers’ health anxiety affects their protection motivation.	Theory illustrates how protection motivation influences smokers’ attitudes toward quitting smoking and, subsequently, their behavioral intention.

The core of PMT is to predict people’s motivation to protect themselves after they perceive threat (Boss et al., 2015). The antecedents of this protection motivation are determined by two levels of appraisals: threat appraisal and coping appraisal (Rogers, 1983). A threat appraisal refers to a person’s appraisal of perceived severity and vulnerability. Perceived severity is an appraisal of the potential severity of an event, and perceived vulnerability is an appraisal of the impact on personal safety of a threat. This impact is predominantly negative. A coping appraisal is a person’s belief that a protective behavior is effective (i.e., response efficacy), belief in successfully performing the protective behavior, and estimated cost of the protective behavior (Floyd et al., 2000; Roozbahani et al., 2020).

In this study, if smokers see GHWLs and think that smoking poses a high degree of threat to them (threat appraisal), and quitting smoking is an effective means of protecting themselves (coping appraisal), smokers will illustrate higher motivation to protect themselves (protection motivation) from harm (Sabzmakan et al., 2018). Furthermore, there is consistent evidence that PMT is suitable for research on quitting and preventing smoking initiation (Yan et al., 2014; Sabzmakan et al., 2018; Lin and Chang, 2021).

In contrast, GHWLs communicate to smokers that they may suffer from the disease in the picture, causing them to worry about health—health anxiety is a widespread mentality (Chen et al., 2021). for the study of GHWLs and quitting smoking, PMT did not consider this aspect (i.e., health anxiety), so it must be solved by introducing E-EPPM. So (2013) combined the EPPM theoretical framework (Witte, 1992), which is most widely used in the field of health, with emotional cognitive evaluation theory (Lazarus, 1991), proposing the E-EPPM.

The E-EPPM incorporates risk perception into a more comprehensive perspective and adds another emotional structure (anxiety) (So, 2013). This study believed that health anxiety is a reasonable and distinct part of emotional arousal during threat appraisal. However, the warning on the cigarette pack tells smokers that they may suffer from illness or get sick in the future, making smokers worry about their health and causing anxiety (Lazard et al., 2018). Consequently, this study introduces the E-EPPM to measure whether health anxiety is the antecedent of smokers quitting.

Furthermore, the TPB is one of the most frequently cited and influential models for predicting human behavior (Ajzen, 2011). The TPB is a theory of rational decision-making that describes what information is processed during deliberative decision-making and proposes that people’s behavior can be predicted most accurately from their intentions—calculated by assessing how much effort they intend to expend in the future (Ajzen, 1991; Pang et al., 2021). One of Ajzen’s (1985) central assertions is that the latent constructs of attitude—positive or negative judgments about performing future behaviors—can predict people’s behavioral intentions. Behavioral intention is the direct cause of the behavior and can be used as an agent to evaluate actual behavior (Ajzen, 1991). Furthermore, TPB has been widely used in behavioral research related to smoking cessation and health risks (Norman et al., 1999; Conner et al., 2002; Tseng et al., 2018; Pang et al., 2021). Consequently, this study introduces TPB into our research framework to measure the positive and negative attitudes and smokers’ behaviors after seeing GHWLs.

### 2.2. Hypothesis development

#### 2.2.1. Core variables of PMT: perceived severity, perceived vulnerability, and response efficacy

According to PMT, the antecedents of protection motivation are determined by two types of appraisals (threat appraisal and coping appraisal) (Boss et al., 2015). Perceived severity in threat appraisal is the degree to which a person believes that a particular threat will cause harm, and vulnerability is the probability that a person considers the corresponding threat will occur. Response efficacy in coping assessment refers to the effectiveness of a particular method considered by the individual (Rogers, 1983; Boss et al., 2015).

This study also proposes that perceived severity, perceived vulnerability, and response efficacy affect protection motivation (Ling et al., 2019; Wang J. et al., 2019; Cummings et al., 2021). Therefore, when smokers see GHWLs, they conduct threat and coping appraisals, leading to a protective motivation to protect themselves by quitting smoking. Accordingly, the following hypotheses are proposed:

H1: Perceived severity has a positive impact on smokers’ protection motivation after seeing GHWLs.

H2: Perceived vulnerability has a positive impact on smokers’ protection motivation after seeing GHWLs.

H3: Response efficacy has a positive impact on smokers’ protection motivation after seeing GHWLs.

#### 2.2.2. Effect of health anxiety on smokers’ protection motivation

The E-EPPM proposed by So (2013) suggests that anxiety is one of the important emotions that people generate through threat perception, vital to causing protective motivation. Therefore, after considering perceived severity and perceived vulnerability, we introduced E-EPPM and considered the role of anxiety. In this study, the threat smokers perceive after seeing GHWLs is predominantly health-related, so we narrowed our perspective to a health-centric perspective, focusing on smokers’ health anxiety. Accordingly, health anxiety is defined as smokers’ worry about their health and produces negative emotions after seeing GHWLs (Zou et al., 2021).

Similarly, Park and Oh (2021) verified the causal relationship between anxiety and prevention behavior in a study on anxiety and threat prevention. Furthermore, Knowles and Olatunji (2021) verified the causal relationship between anxiety and safety behavior in a study on COVID-19. Based on these research results, this study proposes the following hypothesis:

H4: Health anxiety has a positive impact on smokers’ protection motivation after seeing GHWLs.

#### 2.2.3. Effect of protection motivation on smokers’ behavioral intention and attitudes toward quitting smoking

After seeing GHWLs, smokers have the motivation to protect themselves by quitting smoking to prevent tobacco from harming them (Mannocci et al., 2014; Villanti et al., 2014; Sabzmakan et al., 2018). The concept of protection motivation is the core of PMT (Rogers, 1983; Floyd et al., 2000). We explore whether this protection motivation affects people’s actual behavior and results in a positive attitude toward quitting smoking. We assert that after seeing GHWLs, smokers’ protection motivation is positively correlated with their intention to quit smoking, and they will have a positive attitude toward quitting smoking because the most effective way to reduce the harm of tobacco is to quit (van Schayck et al., 2002). Mo et al. (2021) reported that protective motivation is the antecedent of healthy behavior in research on COVID-19. Furthermore, Rogers et al. (2016) found that protection motivation is a key construct influencing health-related attitudes and behaviors in a study of AIDS prevention. Accordingly, the following hypotheses are proposed:

H5: Protection motivation has a positive impact on smokers’ behavioral intention to quit smoking after seeing GHWLs.

H6: Protection motivation has a positive impact on smokers’ attitudes toward quitting smoking after seeing GHWLs.

#### 2.2.4. Attitude on smokers’ behavioral intention to quit smoking

According to TPB, positive or negative attitudes toward performing future behaviors can predict people’s behavioral intentions (Ajzen, 2011). We tend to explore whether these positive or negative attitudes toward quitting smoking will affect their behavioral intention to quit smoking after seeing GHWLs. Pang et al. (2021) reported that people’s attitudes toward recommended methods significantly impacted their behavioral intentions during the COVID-19 pandemic. Similarly, Macy et al. (2012) verified based on the TPB that smokers’ attitudes toward quitting smoking have a significant impact on behavioral intention to quit smoking. In this study, we assert that smokers will have a positive attitude toward quitting smoking after seeing GHWLs (Grigaliunaite and Pileliene, 2017) and that GHWLs will affect their intention to quit smoking. This assertion is consistent with the view proposed by the TPB, so we propose the following hypothesis:

H7: Attitude toward quitting smoking has a positive impact on smokers’ behavioral intention after seeing GHWLs.

## 3. Materials and methods

### 3.1. Research design and measurement items

This study adopted an anonymous cross-sectional survey to conduct an empirical examination of the research hypotheses, which has been widely used in related research (Zeng et al., 2022). The questionnaire consists of three sections. The first section explains the background and purpose of the survey. The second section presents GHWLs to the respondents, including pictures of diseases caused by smoking. GHWLs used in this study were selected from the US Food and Drug Administration in 2011, which were rated as the most effective labels to help smokers understand health effects of smoking (Hammond et al., 2012). In addition, since our research context is China, Chinese annotations were made for the textual information in the tags. Then the respondents were asked 25 items (Table 2) designed to measure seven latent variables: perceived severity, perceived vulnerability, response efficacy, health anxiety, protection motivation, attitude, and behavioral intention. Two questions were added to the second section that asked the respondents to choose “strongly disagree” and “strongly agree” to ensure the authenticity of the responses. Finally, the third section asks about the respondents’ social-demographic information, including gender, age, education, and monthly income, and smoking experience (e.g., pack-year, whether there are friends who smoke, and the monthly cost of tobacco).

**TABLE 2:** Scale development.

Construct	Measurement items	References
Perceived severity (PS)	PS1. The thought of contracting certain diseases from smoking scares me.	Trumbo, 2018; Wang et al., 2018; Pang et al., 2021
	PS2. If I had certain diseases from smoking, then my career would be endangered.	
	PS3. My financial security would be endangered if I had certain diseases from smoking.	
	PS4. If I had certain diseases from smoking, my entire life would change.	
Perceived vulnerability (PV)	PV1. I know that I am at risk of illness from smoking.	Boss et al., 2015
	PV2. I know that I may get sick from smoking.	
	PV3. It is possible that I will get sick from smoking.	
Response efficacy (RE)	RE1. I know that quitting smoking will protect myself.	Boss et al., 2015; Sabzmakan et al., 2018
	RE2. I know that quitting smoking is effective for protection.	
	RE3. I know that quitting smoking is likely to make me healthier.	
	RE4. I know that I will be less likely to get different disease such as heart disease if I quit smoking.	
Health anxiety (HA)	HA1. I felt nervous after seeing GHWLs.	So et al., 2016
	HA2. After seeing GHWLs, I am worried about these diseases due to smoking.	
	HA3. After seeing GHWLs, I feel anxious about my health.	
Protection motivation (PM)	PM1. After seeing GHWLs, I think I need to quit smoking to protect myself.	Boss et al., 2015
	PM2. After seeing GHWLs, I believe that it is necessary to quit smoking to reduce the risk of illness from smoking.	
	PM3. After seeing GHWLs, I believe that it is necessary to quit smoking to reduce the probability of certain diseases from smoking.	
	PM4. I believe that it is necessary for others to quit smoking to reduce the probability of certain diseases from smoking.	
Attitude (AT)	AT1. After seeing GHWLs, I think quitting smoking is beneficial.	Pang et al., 2021; Yao et al., 2021
	AT2. After seeing GHWLs, I think quitting smoking is useful for my health.	
	AT3. After seeing GHWLs, I feel quitting smoking is a wise idea	
Behavioral intention (BI)	BI1. After seeing GHWLs, I plan to quit smoking in the future.	Oh et al., 2020; Pang et al., 2021; Yuen et al., 2021
	BI2. After seeing GHWLs, I came up with a plan to quit smoking.	
	BI3. After seeing GHWLs, I will definitely quit smoking.	
	BI4. After seeing GHWLs, I will not only quit smoking, but also persuade friends around me to quit smoking.	

All items were formulated based on previous studies and the opinions of four relevant experts and scholars in the fields of psychology and addictive behavior management were consulted ensure the validity of the questionnaire. Specifically, we first sent them our original questionnaire and asked for their feedback. After about 2 weeks, we received their comments. Overall, they felt that the wording of some of the questions was too formal and should be made simpler to help respondents understand. In addition, they also suggested adding more demographic survey questions to be used as control variables. Then we carefully prepared responses and revisions based on these suggestions. We then held a webinar with the review panel to discuss the revisions with them in detail and to seek their comments. In this round, the experts were satisfied with the questionnaire and only suggested adding some “attention checkers” to ensure data quality.

Four items (contracting certain diseases from smoking scares me; career will be endangered; financial security will be endangered; entire life will change) of perceived severity were adopted from Trumbo (2018), Wang et al. (2018), and Pang et al. (2021), three items (risk of illness from smoking; may get sick from smoking; It is possible that I will get sick from smoking) were adopted to measure perceived vulnerability (Boss et al., 2015), and four items (quitting smoking will protect myself; quitting smoking is effective for protection; quitting smoking is likely to make me healthier; get different disease if I quit smoking) of response efficacy were adopted from Boss et al. (2015) and Sabzmakan et al. (2018). A three-item scale (I felt nervous after seeing GHWLs; worried about these diseases due to smoking; feel anxious about my health) was chosen from So et al. (2016) to detect health anxiety, and protection motivation (I need to quit smoking to protect myself; it is necessary to quit smoking to reduce the risk of illness from smoking.; it is necessary to quit smoking to reduce the probability of certain diseases from smoking; it is necessary for others to quit smoking to reduce the probability of certain diseases from smoking) was measured by four items (Boss et al., 2015). Three items (quitting smoking is beneficial; quitting smoking is useful for my health; quitting smoking is a wise idea) of attitude were adopted from Pang et al. (2021) and Yao et al. (2021), and respondents’ behavioral intention was measured by four items (I plan to quit smoking in the future; I came up with a plan to quit smoking; I will definitely quit smoking; I will not only quit smoking, but also persuade friends around me to quit smoking) from Oh et al. (2020), Pang et al. (2021), and Yuen et al. (2021). A seven-point Likert scale (1 = “strongly disagree” to 7 = “strongly agree”) was adopted to evaluate these items.

A pre-test was first conducted for respondents to ensure that the final survey results are valid and appropriate. The 25 respondents in the pre-test indicated that they fully understood the purpose and content of the survey (including the warning content of GHWLs). However, three respondents proposed that the description of some questions was not clear enough. For example, respondents said that there are some objections in the original formulation of “PS2: If I had certain diseases from smoking, then my work would be endangered,” which measures perceived severity. Because there were respondents who were not employed at the time of the survey. Hence, we modified the PS2 to read, “If I had certain diseases from smoking, then my career would be endangered” to make the measuring instrument widely applicable. We resubmitted the revised questionnaire to 25 pre-test respondents. After confirming that there was no ambiguity, we use it for final data collection.

Given that structural equation modeling (SEM) is a widely adopted method used to analyze cross-sectional data collected by surveys and experiments (Jiang et al., 2021; Fang et al., 2023; Pang et al., 2023; Su et al., 2023; Xing et al., 2023), this study uses SEM to validate hypotheses, test their reliability and discriminant validity by confirmatory factor analysis (CFA). In addition, SEM is divided into Covariance-based technique (CB-SEM) and Partial Least Square (PLS-SEM). The model of this study is theory-based and requires data for interpretation, which is more suitable for CB-SEM, as PLS is more suitable for exploratory research in the early stages of theoretical development (Hair et al., 2014).

### 3.2. Data collection

The survey was administered on Wen Juan Xing, a commonly used data-collection platform for cross-sectional research (Tao et al., 2021). The survey was conducted in China which was written in Chinese and fully referred to the opinions of relevant experts from the department of Chinese language and literature to ensure a complete understanding of each item. Take a random sample method to conduct surveys to ensure that our surveys can be generalized to a wider range of people. Smokers located in China who are currently smoking were included in the study sample. People with mental illness and dyslexia are excluded. We commissioned Wen Juan Xing to collect samples in accordance with the required methodology and inclusion/exclusion criteria. The survey was conducted from March 2 to June 25, 2021; after excluding invalid surveys with incorrect screening items and too-short response times, 547 surveys were considered for analysis (84.2% conversion rate). In addition, as recommended by Kline (1998), a sample size of more than 200 is required when using SEM analysis. In our research, a total of 547 surveys are analyzed, and the stability of the SEM analysis was not affected.

In this study, Harman’s single factor test proposed by Podsakoff et al. (2003) was performed to examine the possibility of common method variance. The results demonstrate that the total variance of the single factor model is 35.65% (<40%). Thus, common method bias is not an issue in our data. Furthermore, with the method proposed by Armstrong and Overton (1977) and Chaudhuri and Holbrook (2001), the non-response bias is checked by return date, indicating no significant difference between the two groups.

## 4. Results

### 4.1. Demographic statistics and smoking experience

The respondents’ demographics are presented in Table 3. The sample includes 304 males and 243 females, accounting for 55.6 and 44.4%—a slightly higher proportion of males than females. A total of 225 (41.1%) respondents were 20–29 years old, 204 (37.3%) were 30–39 years old, 67 (12.2%) were 40–49 years old, and 51 (9.3%) were over 50 years old. The majority of the interviewees were young and middle-aged people between 20 and 40 years old. The respondents also have a relatively high level of education: 62 (11.3%) graduated from high school, 443 (81%) are enrolled or graduated from university, and the remaining 42 (7.7%) have a higher degree.

**TABLE 3:** Respondent demographics.

Items	Category	Frequency	Percentage (%)
Gender	Male	304	55.6
	Female	243	44.4
Age (years)	20–29	225	41.1
	30–39	204	37.3
	40–49	67	12.2
	>50	51	9.3
Education	High school or below	62	11.3
	Undergraduate	241	44.1
	Bachelor	202	36.9
	Postgraduate or above	42	7.7
Money spent on tobacco per month (CNY) (1 USD = 6.34 CNY^a^)	<100	137	25.0
	100–500	144	26.3
	501–1,000	114	20.8
	>1,000	152	27.8
	>5,000	23	6.4
Pack-year^b^	<1	139	25.4
	1–5	138	25.2
	6–10	79	14.4
	11–15	141	25.8
	>15	50	9.1
The number of friends who are smokers	None	41	7.5
	<3	160	29.3
	4–6	191	34.9
	7–9	146	26.7
	>9	9	1.6
			*N* = 547

^a^US dollar to Chinese Yuan conversion—last updated Feb 21, 2022, 21:26 UTC. ^b^Pack-year is the product of number of packs smoked per day and number of years smoked.

Regarding the average monthly expenditure on smoking, 137 people (25%) spent less than 100 yuan, 144 (26.3%) spent 100–500 yuan, and 114 (20.8%) spent 501–1,000 yuan, 152 people (27.8%) spent more than 1,000 yuan, and 23 (2.3%) spent more than 5,000 yuan. In terms of pack-year, 139 people (25.4%) had smoked for less than 1 pack-year, 138 people (25.2%) had smoked for 1–5 pack-year, 79 people (14.4%) had smoked for 6–10 pack-year, 141 people (25.8%) had smoked for 11–15 pack-year, and 50 people (9.1%) had smoked for more than 15 pack-year. When observing the number of smokers among friends, there were 41 (7.5%) non-smokers among friends, 160 (29.3%) with less than 3 smokers among friends, 191 (34.9%) with 4–6 smokers among friends, 146 (26.7%) with 7–9 smokers among friends, and 9 (1.6%) with more than 9 smokers among friends. most of them are also friends who smoke around the smoker.

### 4.2. Measurement model assessment

Confirmatory factor analysis was conducted to evaluate model fit, reliability, and validity. The results are presented in Table 4. As suggested by previous studies (Bentler and Bonett, 1980; Browne and Cudeck, 1992), χ^2^/df, comparative fitting index (CFI), Tucker-Lewis index (TLI), root mean square error (RMSEA), and standardized root mean square residual (SRMR) were selected to evaluate the model fit. Hair et al. (2010) suggest that (1) χ^2^/df should be between 1 and 3, and the smaller the better. χ^2^/df, the better the model fit; and (2) the closer the CFI is to 1, the better the model fit, generally, a value greater than 0.9 is a good fit. TLI is a type of comparative fitting index (Marsh et al., 1988), and like CFI, the closer to 1, the better, and generally the model fit is better when it is greater than 0.9. RMSEA generally recommends less than 0.05, with larger values indicating a better fit between the model and the data (Browne and Arminger, 1995). SRMR less than 0.08 is generally acceptable, and less than 0.05 makes a good model fit (Joreskog and Sorbom, 1989). As presented in Table 4, our measurement model exhibits good model fit because the indices are all within the cut-off range (χ^2^/df = 1.931, *p* < 0.05, CFI = 0.961, TLI = 0.955, RMSEA = 0.041, and SRMR = 0.035) (Bentler and Bonett, 1980; Hu and Bentler, 1999).

**TABLE 4:** Confirmatory factor analysis results.

Construct	Item	λ	AVE	CR
Perceived severity (PS)	PS1	0.725	0.608	0.861
	PS2	0.774		
	PS3	0.828		
	PS4	0.789		
Perceived vulnerability (PV)	PV1	0.712	0.546	0.828
	PV2	0.769		
	PV3	0.720		
	PV4	0.752		
Response efficacy (RE)	RE1	0.729	0.561	0.836
	RE2	0.803		
	RE3	0.734		
	RE4	0.726		
Health anxiety (HA)	HA1	0.756	0.580	0.847
	HA2	0.765		
	HA3	0.720		
	HA4	0.804		
Protection motivation (PM)	PM1	0.802	0.674	0.892
	PM2	0.825		
	PM3	0.849		
	PM4	0.806		
Attitude (AT)	AT1	0.717	0.565	0.838
	AT2	0.739		
	AT3	0.776		
	AT4	0.772		
Behavioral intention (BI)	BI1	0.750	0.533	0.820
	BI2	0.691		
	BI3	0.729		
	BI4	0.750		

Model fit indices: χ^2^/df = 1.931 (*p* < 0.05, df = 329); CFI = 0.961; TLI = 0.955; RMSEA = 0.041; SRMR = 0.035.

Simultaneously, all seven structures have a composite reliability (CR) above 0.7 (Table 4), confirming the model’s reliability (Hair et al., 2010; Wang et al., 2018). This reflects that all questions in each latent variable can interpret that latent variable consistently. A convergent validity test was conducted by analyzing the average variance extracted (AVE) and the standardized factor loading (Hair et al., 2010; Wang et al., 2018). AVE can be used to measure convergence validity, generally greater than 0.5 to indicate that the observed variable can explain enough variation in this latent variable. All standardized factor loadings and AVE values exceeded the recommended value of 0.5, indicating good convergent validity (Table 4).

Regarding the discriminant validity between various constructs, the AVE value of each structure is greater than the square of the correlation value with other structures, as indicated by Table 5. Thus, the discriminant validity of the model is verified (Hair et al., 2010).

**TABLE 5:** Average variance extracted (AVE), correlations, and squared correlations of constructs.

	PS	PV	RE	HA	PM	AT	BI
PS	**0.608^a^**	0.154^c^	0.194	0.264	0.442	0.434	0.384
PV	0.393^b^	**0.546**	0.090	0.147	0.232	0.072	0.141
RE	0.440	0.300	**0.561**	0.144	0.250	0.368	0.184
HA	0.514	0.384	0.380	**0.580**	0.366	0.183	0.297
PM	0.665	0.482	0.500	0.605	**0.674**	0.350	0.403
AT	0.659	0.269	0.607	0.428	0.592	**0.565**	0.464
BI	0.620	0.375	0.429	0.545	0.635	0.681	**0.533**

^a^AVE values are along main diagonal (bold values). ^b^Correlations between constructs are below main diagonal. ^c^Squared correlations between the constructs are above main diagonal.

### 4.3. Structural model assessment

**FIGURE 3:**
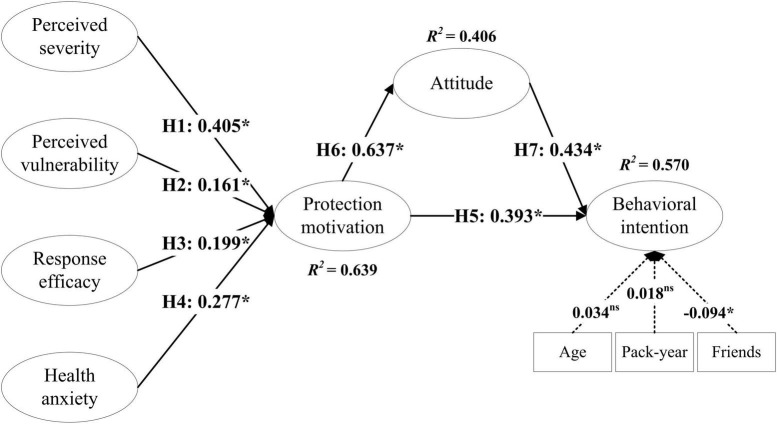
Results of structural model analysis. *p < 0.05. Standardized coefficients are displayed in the path.

Structural equation modeling analysis was used to examine the proposed hypotheses, with the results graphically depicted in Figure 3. As expected, the results suggest a good model fit (χ^2^/df = 2.062, *p* < 0.05, CFI = 0.943, TLI = 0.937, RMSEA = 0.044, and SRMR = 0.057). variables including age, pack-year, and friends were considered in the model to control the marginal effects on smokers’ behavioral intentions to quit smoking.

There is a significant positive correlation between perceived severity, perceived vulnerability, response efficacy, health anxiety, and protection motivation at the 5% significance level. Standardized coefficients of 0.405, 0.161, 0.199, and 0.277 indicate that Hypotheses H1, H2, H3, and H4 are all accepted.

The results in Figure 3 also reveal that protection motivation has a statistically positive effect on behavioral intention (β = 0.393, *p* < 0.05), indicating that Hypothesis H5 is accepted. The positive effect of protection motivation on attitude is also significant (β = 0.637, *p* < 0.05), indicating that Hypothesis H6 is accepted. Finally, it was verified that attitude had a significant positive effect on behavioral intention (β = 0.434, *p* < 0.05), indicating that Hypothesis H7 was accepted.

Moreover, when control variables (age, pack-year, and friends) were compared with smokers’ behavioral intentions, age and pack-year were not statistically significant. Previous studies have found that smoking cessation behavior is more susceptible to peer influence during adolescence (Jones et al., 2004). Studies even found that smokers’ friends have greater influence than parents on smokers with higher self-perceived vulnerability, such as asthmatics (Van Zundert et al., 2008).

### 4.4. Effects analysis

This study examines the influence of exogenous variables on endogenous variables (Table 6). In the theoretical model depicted in Figure 3, protection motivation and attitude are added to the model as intermediary variables.

**TABLE 6:** Direct, indirect, and total effects.

Exogenous (i)	Endogenous (j)		
	PM (1)	AT (2)	BI (3)
**Direct effect (a)**			
PS (1)	0.405	–	
PV (2)	0.161	–	
RE (3)	0.199	–	
HA (4)	0.277	–	
PM (5)	–	0.637	0.393
AT (6)	–	–	0.434
**Indirect effect (b)**			
PS (1)	–	0.258	0.271
PV (2)	–	0.103	0.108
RE (3)	–	0.127	0.133
HA (4)	–	0.177	0.186
PM (5)	–	–	0.277
AT (6)	–	–	–
**Total effect (c)**			
PS (1)	0.405	0.258	0.271
PV (2)	0.161	0.103	0.108
RE (3)	0.199	0.127	0.133
HA (4)	0.277	0.177	0.186
PM (5)	–	0.637	0.669
AT (6)	–	–	0.434

The direct effect of PV on PM is expressed as a21.

According to the test results of direct effects, perceived severity (a11 = 0.405), perceived vulnerability (a21 = 0.161), response efficacy (a31 = 0.199), and health anxiety (a41 = 0.277) used in PMT and the E-EPPM all have a direct impact on protection motivation. Perceived severity has the most direct impact among them. Attitude is influenced most directly by the self-protection motivation (a52 = 0.637), i.e., self-protection motivation significantly promotes the smoker’s attitude toward quitting. Furthermore, attitude (a63 = 0.434) has a direct impact on behavioral intention, and protection motivation (a53 = 0.393) has a direct impact on behavioral intention.

In terms of indirect effects on attitude, perceived severity (b12 = 0.258) has the largest indirect impact, followed by heat anxiety (b42 = 0.177), response efficacy (b32 = 0.127) and perceived vulnerability (b22 = 0.103). In terms of indirect effects of behavioral intention, protection motivation (b53 = 0.277) has the largest indirect effect, followed by perceived severity (b13 = 0.271), health anxiety (b43 = 0.186), response efficacy (b33 = 0.133), and perceived vulnerability (b23 = 0.108).

The combined total effect of these factors indicates that protection motivation (c53 = 0.669) has the greatest impact on behavioral intention followed by attitude (c63 = 0.434). Protection motivation has the greatest impact on attitude (c52 = 0637), and perceived severity has the greatest impact on protection motivation (c11 = 0.405). Consequently, protection motivation is a vital intermediary when smokers take action to quit smoking.

## 5. Discussion

It is human nature to respond to external dangers with protective mechanisms, especially in more vulnerable areas. Our research results show that perceived severity, perceived vulnerability, response efficacy have a significant positive effect on protection motivation. When smokers view the GHWL pictures, they are constantly reminded of the severe consequences of smoking and the vulnerability of their bodies. The higher the probability of these severe consequences, the easier it will be for smokers to develop self-protection ideas, including quitting. Furthermore, PMT suggests factors to respond to the evaluation. Response efficacy refers to the effectiveness of recommended behaviors in response to threats. A reflective evaluation is a type of coping appraisal of PMT. By considering the effects, smokers will take protective measures beneficial to themselves and adopt risk control behaviors, consistent with PMT-based results in previous studies (Ling et al., 2019; Wang J. et al., 2019; Cummings et al., 2021). Furthermore, research results show that smokers who realize that smoking is harmful to their health will feel anxiety, triggering protection motivation. They will take measures like quitting to protect themselves, consistent with the results of E-EPPM in previous studies (So, 2013; Park and Oh, 2021). Among the four influencing factors, perceived severity has the greatest positive impact on protective motivation, indicating that smokers are more likely to receive the effects of smoking risk and produce protective awareness such as quitting smoking, followed by health anxiety, response efficacy, and perceived vulnerability.

Meanwhile, the results demonstrate that smokers’ protection motivation has a positive impact on attitude and behavioral intention. After seeing GHWL, the apparent protection consciousness of smokers will promote the behavior of smoking cessation. Smokers’ attitudes toward smoking cessation indicate their behavior: the more aware smokers are of their own self-protection, the more supportive they are of their desire to quit smoking.

Simultaneously, the verification results of this study demonstrate that attitudes have a positive correlation with smoking cessation behaviors. As verified by TPB, positive smoking cessation attitudes directly affect smoking cessation behaviors. These conclusions are consistent with the results of previous studies (Macy et al., 2012; Sabzmakan et al., 2018). Consequently, when trying to guide smokers to quit smoking, it is necessary to strengthen smokers’ personal self-protection awareness. This can be achieved by focusing on guiding and establishing changes in smokers’ attitudes toward the severe consequences of smoking.

This study covers a broad range of topics; compared to existing studies (Wong et al., 2015), age no longer influences smoking cessation behavior. The number of friends has a negative effect on the intention to quit smoking. Consistent with previous studies (Jones et al., 2004; Dunbar et al., 2021), this finding implies that the more friends who smoke around a smoker, the more difficult it will be to quit (Schwartz et al., 2022; Vallarta-Robledo et al., 2022). Furthermore, in this study, pack-year has no significant effect on behavioral intentions, contrary to the conclusion of the previous study (Turner et al., 2005). Compared with smoking frequency, there are also more pronounced factors that influence smokers’ willingness to quit smoking, which is the purpose of this study. According to this study, the theoretical structure also predicts smokers’ willingness to quit smoking more accurately than demographic characteristics.

## 6. Conclusion

This study explores the effect of GHWLs on smokers’ smoking cessation behavior through theoretical derivation and empirical testing. We built a theoretical model based on protection motivation theory, an extension of the extended parallel process model, and the theory of planned behavior. A total of 547 smokers’ data is used to test research hypotheses through structural equation models. The findings indicate perceived severity, perceived vulnerability, response efficacy, and health anxiety have a significant impact on smokers’ protection motivation. Furthermore, smokers’ protection motivation has a significant effect on quitting behavior and can indirectly influence behavioral intention through smokers’ attitude toward GHWLs. Based on the above findings, the theoretical contributions and managerial implications made by this study are as follows.

### 6.1. Theoretical contributions

This study makes several significant theoretical contributions to the literature. First, it enriches the literature on public health protection measures (i.e., GHWLs) and smokers’ behavioral intention to quit smoking. Smoking is still the preventable behavior that poses the greatest threat to public health globally, so the WHO introduced the FCTC in 2003, proposing the use of GHWLs as a means of warning and protection for public health. GHWLs can tell smokers the threats they will face in the most intuitive form of pictures. This research explored the potential psychological factors that smokers generate quit intention after seeing GHWLs to examine the influence of GHWLs on smokers’ behavioral intention to quit.

Second, based on PMT, this study combined the TPB and the E-EPPM to construct a new theoretical model to explain the antecedents of smokers’ intentions to quit smoking after seeing GHWLs. This theoretical model can explain the impact of health advice on people’s protection behaviors by exploring the influence of GHWLs on smokers’ protection intentions and attitudes. Furthermore, this study found that more positive attitudes toward quitting smoking and higher protection motivation promote the behavioral intention to adopt this health advice. If GHWLs make smokers understand the severity of smoking and their own vulnerability, their motivation to protect themselves will be strengthened. Protection motivation can also be enhanced by producing health anxiety and recommending appropriate methods. Moreover, the higher motivation of smokers for protection also promotes a more positive attitude toward quitting smoking, consistent with previous research results (Sabzmakan et al., 2018; Pang et al., 2021; Yao et al., 2021; Yuen et al., 2021).

Third, this study has enriched PMT and the TPB. This study evaluated the indirect influence of the basic structure of PMT on behavioral intentions when protection motivation was used as a mediating variable. Under the adequate mediation of protection motivation, the three structures of PMT (perceived severity, perceived vulnerability, and response efficacy) and the health anxiety of E-EPPM have an indirect impact on people’s protection behaviors. Furthermore, attitudes mediated the relationship between protection motivation and behavioral intention. Total effect analysis revealed that smokers’ intention to quit smoking was most strongly affected by protection motivation, followed by attitude, perceived severity, health anxiety, response efficacy, and perceived vulnerability. Therefore, the mediating role of protection motivation and attitudes better reflects the logical reasoning of people taking protection behaviors, leading to their final intentions.

### 6.2. Policy implications

Because this study analyzed how GHWLs affect smokers’ intention to quit, our results also provide some management implications. First, the visual threat perception of GHWLs can tell smokers more intuitively that smoking may cause serious diseases. Given the significant impact of perceived vulnerability and perceived severity, GHWLs must play the health warning role. The core function of GHWLs is to convey to smokers that smoking causes serious diseases and what those are (Ratih and Susanna, 2018). Therefore, countries that have implemented the GHWLs policy can expand the warning information of GHWLs, enrich the disease pictures of GHWLs (e.g., on various organ diseases caused by smoking), and cooperate with more abundant text warnings to warn the public about the harm of smoking. In countries that have not yet implemented a GHWLs policy, this policy should be implemented as soon as possible to warn the public more effectively about the dangers of smoking. This requires borrowing the power of the institution and legislating to solve the problem (Wang et al., 2023).

Second, after smokers see GHWLs, their response efficacy and health anxiety have a significant impact on protection motivation. Accordingly, GHWLs can motivate smokers to generate protection motivation by causing smokers to produce health anxiety and informing them of the effectiveness of quitting smoking. This finding is enlightening for management. For example, a comprehensive implementation of the GHWLs policy in a country or region, labeling GHWLs on all cigarette packs and making them exist for a long time, popularizes and normalizes GHWLs. Furthermore, amplifying the GHWLs on the cigarette pack to make it cover the cigarette pack as much as possible can prevent smokers from subtly avoiding the impact of GHWLs on the pack. Finally, because quitting smoking is the most effective way of protecting themselves, it is necessary to continuously inform the public that “quitting smoking is the most effective means” in cigarette packs or other public channels.

Third, considering the significant impact and mediating effects of protection motivation and attitude toward quitting smoking, it is necessary to strengthen smokers’ protection motivation by enhancing the three structures of PMT and the health anxiety of E-EPPM. Thus, smokers should be told about the benefits of quitting smoking in cigarette packs and other mass media that smokers can access (e.g., reduce the risk of myocardial infarction, lung cancer and coronary heart disease, improve blood circulation, and enhance immunity) (Godtfredsen and Prescott, 2011). Moreover, regulators must inform smokers that they will not lose anything if they quit smoking (e.g., if you quit smoking, you will be empty, and you will lose fun).

### 6.3. Limitations and recommendations

This study has some limitations in providing directions for follow-up research. First, this study discussed the impact of GHWLs on smokers’ intention to quit smoking at smokers’ psychological levels and discussed the implementation of the GHWLs policy and how to improve it. However, given available data, this study was conducted in China, where GHWLs policies are not implemented. After observing GHWLs for Chinese smokers, the psychological activities influenced by them were analyzed. Although the research results support that this policy should be implemented in China, follow-up research encourages comparison with other countries that have implemented this policy. Furthermore, this study used PMT, combined with E-EPPM and TPB, and produced corresponding findings. Nevertheless, this study still encourages researchers to explore the effects of GHWLs on smokers’ smoking cessation behavior from a broader perspective. Moreover, the majority of our research sample is under 40 years old and highly educated, and future study subjects are encouraged to be over 40 years old and have low education for cross-validation. Finally, because young smokers typically do not experience smoking-related illness for decades to come, their perceived threat tends to be greatly reduced (Carroll et al., 2023). The time perspective of younger smokers was not considered in this study, and it is suggested that younger smokers (under 20 years of age) could be considered in future studies.

## Data availability statement

The raw data supporting the conclusions of this article will be made available by the authors, without undue reservation.

## Author contributions

QP: conceptualization, methodology, writing—original draft, and resources. LW: conceptualization and writing—original draft. JY: conceptualization and writing—review and editing. KY: methodology and writing—review and editing. MS: resources and methodology. MF: conceptualization, methodology, software, and resources. All authors contributed to the article and approved the submitted version.
